# Disease Modeling of Pituitary Adenoma Using Human Pluripotent Stem Cells

**DOI:** 10.3390/cancers14153660

**Published:** 2022-07-27

**Authors:** Ryusaku Matsumoto, Hidetaka Suga, Hiroshi Arima, Takuya Yamamoto

**Affiliations:** 1 Center for iPS Cell Research and Application, Kyoto University, Kyoto 606-8507, Japan; r.matsumoto@cira.kyoto-u.ac.jp (R.M.); takuya@cira.kyoto-u.ac.jp (T.Y.); 2 Department of Endocrinology and Diabetes, Nagoya University Graduate School of Medicine, Nagoya 466-8550, Japan; arima105@med.nagoya-u.ac.jp; 3 Institute for the Advanced Study of Human Biology (ASHBi), Kyoto University, Kyoto 606-8501, Japan; 4 Medical-Risk Avoidance Based on iPS Cells Team, RIKEN Center for Advanced Intelligence Project (AIP), Tokyo 103-0027, Japan

**Keywords:** pituitary tumor, pluripotent stem cell, organoid, ACTH-producing adenoma, GH-producing adenoma, USP8, GNAS, CRISPR/Cas9

## Abstract

**Simple Summary:**

Pituitary adenoma pathophysiology has been studied mainly using murine cell lines, animal models, and pituitary tumor samples. However, the lack of human pituitary cell line is a significant limiting factor in studying the molecular mechanisms of human pituitary tumors. Recently, pituitary induction methods from human-induced pluripotent stem cells (hiPSCs) have been established. These methods can induce human pituitary hormone-producing cells that retain physiological properties. hiPSCs in which tumor-causing gene mutations are introduced using genome-editing techniques, such as CRISPR/Cas9 systems, provide great opportunities to establish in vitro human pituitary adenoma disease models. The models will be a novel platform to discover novel drugs and investigate tumorigenesis and pathophysiology. The purpose of this review is to provide an overview of the applications of iPSCs for pituitary and neoplastic disorder research and genome-editing technologies to create strategies for developing pituitary adenoma models using iPSCs.

**Abstract:**

Pituitary adenomas are characterized by abnormal growth in the pituitary gland. Surgical excision is the first-line treatment for functional (hormone-producing) pituitary adenomas, except for prolactin-producing adenomas; however, complete excision is technically challenging, and many patients require long-term medication after the treatment. In addition, the pathophysiology of pituitary adenomas, such as tumorigenesis, has not been fully understood. Pituitary adenoma pathophysiology has mainly been studied using animal models and animal tumor-derived cell lines. Nevertheless, experimental studies on human pituitary adenomas are difficult because of the significant differences among species and the lack of reliable cell lines. Recently, several methods have been established to differentiate pituitary cells from human pluripotent stem cells (hPSCs). The induced pituitary hormone-producing cells retain the physiological properties already lost in tumor-derived cell lines. Moreover, CRISPR/Cas9 systems have expedited the introduction of causative gene mutations in various malignant tumors into hPSCs. Therefore, hPSC-derived pituitary cells have great potential as a novel platform for studying the pathophysiology of human-specific pituitary adenomas and developing novel drugs. This review presents an overview of the recent progresses in hPSC applications for pituitary research, functional pituitary adenoma pathogenesis, and genome-editing techniques for introducing causative mutations. We also discuss future applications of hPSCs for studying pituitary adenomas.

## 1. Introduction

The pituitary gland plays a pivotal role in maintaining physical homeostasis by releasing pituitary hormones into the blood stream. It comprises two different entities, the anterior and posterior lobes, which have different embryonic origins. The anterior pituitary lobe produces adrenocorticotropic hormone (ACTH), thyroid-stimulating hormone (TSH), growth hormone (GH), prolactin (PRL), luteinizing hormone (LH), and follicle-stimulating hormone (FSH). Pituitary adenoma is an abnormal growth in the pituitary gland arising from single precursor-cell expansion [1]. Pituitary tumors are the third most common intracranial tumors, accounting for up to 15% primary brain tumors [2]. Although most pituitary tumors are benign and rarely metastasize to other organs, they frequently invade surrounding tissues, such as the skull base and cavernous sinus, and over-secrete pituitary hormones that affect other endocrine organs and cause various symptoms. Pituitary adenomas are roughly grouped into two types: functional (hormone-producing) and non-functional (non-hormone-producing) pituitary adenomas. This review focuses on functional pituitary adenomas because functional pituitary adenomas are often clinically refractory to medical treatment, and it is desirable to develop novel treatment modalities and elucidate tumorigenesis mechanisms. Currently, several treatment options are available for each pituitary adenoma type. Surgical resection is the first-line treatment for ACTH-, TSH-, and GH-producing adenomas (ACTHoma, TSHoma, and GHoma) [3,4,5,6]. However, total resection of pituitary adenomas is technically challenging in some patients because of the anatomic inaccessibility of the pituitary gland and the proximity of the brain and vascular structures. Therefore, these patients remain over-secreting and require persistent medical therapy after tumor resection. Furthermore, normal pituitary gland over-resection causes secondary hypopituitarism, which significantly impairs quality of life after treatments. On the other hand, PRL-producing adenoma (PRLoma) also had required surgical resection; however, cabergoline, a dopamine agonist, replaced surgical treatment and became the first-line treatment because it normalizes serum PRL levels in almost all patients [7]. Other functional pituitary adenomas also warrant novel medical treatment as substitutes for surgical treatments. Understanding pituitary adenoma pathophysiology is essential for novel drug development. Present studies on pituitary adenoma pathophysiology mainly use animal models, such as mice and rats and rodent tumor-derived cell lines, both of which are far from human pathology. Although primary cell cultures of human pituitary tumor samples can be used for short-term studies, however, clinical opportunities for obtaining pituitary tumor samples are relatively limited. In addition, immortalized human pituitary cell lines have not been successfully established. Therefore, there have been several difficulties in studies of pituitary adenoma pathogenesis. Recently, several studies have developed pituitary induction methods using human pluripotent stem cells (hPSCs). The induced pituitary cells have physiological properties lost in rodent tumor-derived cell lines. These cells produce and release pituitary hormones in response to hypothalamic-stimulating hormones. Moreover, these induced cells possess feedback systems known as normal pituitary functions; when the cells are treated with hormones produced from the target organs, they cease to secrete pituitary hormones. Due to these characteristics, hPSC-derived pituitary cells may be used as a novel disease model for pituitary disorders.

Furthermore, in the past decade, genome-editing technologies using CRISPR/Cas systems have become a standard in many laboratories because of their facilities, effective and specific editing properties, and low cost. CRISPR/Cas systems introduce causative mutations for various malignant tumors into hPSCs. Combining hPSC-derived pituitary hormone-producing cells and genome-editing technologies has great potential to produce a breakthrough in pituitary tumor research. In this review, we provide an overview of pituitary adenoma pathogenesis, pituitary induction methods using hPSCs, and CRISPR/Cas genome-editing systems. We also discuss future applications of hPSCs in the study of pituitary adenomas.

## 2. Pituitary Adenoma Pathogenesis

Functional pituitary adenomas may over-secrete pituitary hormones, such as PRL, GH, ACTH, and rarely LH, FSH, and TSH. Clinically, PRLoma, GHoma, and ACTHoma are the most frequent in this order. In most PRLomas, hyperprolactinemia can be controlled with the dopamine agonist cabergoline [7]. Thus, this review focuses on ACTHoma and GHoma for which its response rates to medical therapies are inadequate [6,8,9].

### 2.1. ACTH-Producing Adenoma

ACTHoma causes Cushing’s disease, which is associated with increased morbidity and mortality mainly due to cardiovascular events [10]. Most ACTHomas occur sporadically, except for few familial Cushing’s diseases such as multiple endocrine neoplasia type 1 (MEN1), MEN4, and familial isolated pituitary adenomas (FIPAs). Germline mutations associated with these hereditary syndromes have been identified in the *MEN1* [11], *CDKN1B* [12], and aryl hydrocarbon receptor-interacting protein (*AIP*) genes [13]. These genetics of familial Cushing’s diseases are well reviewed in the other article [14].

The most important cause for sporadic ACTHomas is somatic mutations within the ubiquitin-specific protease 8 (*USP8*). Whole-exome sequencing studies of sporadic patients revealed that almost 50% ACTHomas have somatic mutations in *USP8* [15,16], and mutations in this gene have been detected in 40–60% of sporadic ACTHoma cases [17,18]. All point mutations were located within the 14-3-3 binding motif of USP8. These mutations impair the interaction between 14-3-3 and USP8 and enhance its cleavage by a specific protease, thus increasing USP8 deubiquitylation activity. One of the important deubiquitination substrates of USP8 is the epidermal growth factor receptor (EGFR) [19]. EGF is a major mitogenic and differentiation factor of particular importance in ACTHoma pathophysiology. EGFR is frequently overexpressed in ACTHomas and is essential for the synthesis of proopiomelanocortin (POMC), an ACTH precursor [20]. Mutations clustered in the 14-3-3 binding motif enhanced USP8 catalytic activity, thus increasing EGFR deubiquitination and sustaining EGF signaling. These data indicate that mutations in USP8 cause ACTHoma via EGFR-signaling activations [16].

Other reports have tried identifying causative genes for sporadic ACTHomas using next-generation sequencing. The whole-exome sequencing of ACTHomas with wild-type *USP8* identified driver mutations within *USP48*, *BRAF*, and *TP53* genes [21,22]. The *BRAF* p.V600E mutation has been identified in several malignant tumors, including melanoma, papillary thyroid carcinomas, colon cancer, gliomas, and papillary craniopharyngiomas. This mutation increases cell proliferation due to the MAPK pathway activation. *TP53* is a well-known tumor suppressor gene that plays vital roles in DNA repair, cellular senescence, and apoptosis.

In addition to the genetic mutations involved in ACTHoma tumorigenesis described above, genes with characteristic expression changes in ACTHoma and genes involved in the pathogenesis of the disease have been reported. ACTHomas are characterized by the aberrant expression of proteins involved in cell-cycle regulation, proliferation, growth, and differentiation. The cyclin E and cyclin-dependent kinase (CDK)2 complex driving the G1/S transition is overexpressed, and the p27/Kip1 tumor-suppressor protein inhibiting the cyclin E/CDK2 complex is reduced in ACTHomas [23,24]. Another gene associated with aggressive ACTHoma is CDK5 and ABL1 enzyme substrate 1 (CABLES1), a tumor suppressor gene that negatively regulates the cell cycle by inactivating several CDKs. CABLES1 is expressed in normal human pituitary cells, but its expression is strongly downregulated in ACTHomas. CABLES1 loss downregulates the cell cycle inhibitors p21/Cip1 and p27/Kip1 by destabilizing the inhibitors, contributing to ACTHoma development [25].

### 2.2. GH-Producing Adenoma

GHoma causes acromegaly in adults and gigantism in childhood and adolescence [26]. The most important cause of GHoma is somatic gain-of-function mutations in the α-subunit of the stimulatory Gα protein gene (*GNAS*) at codon 201 and 207. Although the frequency of *GNAS* mutation in GHomas varies depending on the ethnicity of patients, it has been reported that 20 to 50% of sporadic GHomas are associated with this mutation [27,28,29,30]. *GNAS* mutations constitutively activate adenylate cyclase, which converts ATP to cAMP. Increased intracellular cAMP concentration activates protein kinase A and mitogenic signaling in GH-producing cells [31]. Although most GHomas are sporadic, a few GHomas are associated with germline defects. Pituitary adenomas, including GHoma, occur as components of familial multiple neoplasia syndromes, such as MEN1, Carney’s complex, and MEN4 [12,32,33]. Furthermore, germline mutations in the *AIP* and X-chromosomal microduplication cause GHomas [34,35]. These hereditary GHomas are reviewed in another review article [36].

Even after testing the above causal genes for GHoma, more than half of GHomas have unknown etiologies [27]. To date, several studies have reported the results of next-generation sequencing (NGS) analysis in GHomas, including whole-exome and whole-genome sequencing, to discover novel causal gene defects and unveil the etiologies. Valimaki et al. performed whole-genome sequencing of 12 GHomas and their corresponding blood samples [37], identified 129 somatic single-nucleotide variants (SNVs) per tumor, and found only 2.3 SNVs on average per tumor in coding regions. The oncogenic *GNAS* mutation (p.Arg201Cys) was the only recurrent somatic event. Recently, a comprehensive genome-wide analysis of functional pituitary tumors also reported that only *GNAS* mutations were recurrent in GHomas, consistent with previous reports [38]. In association with GHoma due to X chromosome microduplication (X-LAG), G protein-coupled receptor 101 (*GPR101*) was identified as the causative gene of the duplicated region, and it was reported that GPR101 mutations are also identified in sporadic GHomas [35].

As another possible cause of GHoma tumorigenesis, glucose-dependent insulinotropic polypeptide receptor (GIPR) overexpression was described in *GNAS* mutation-negative GHomas [39,40]. GIPR is activated by GIP, which is secreted by the K cells in the duodenum, and activates the cAMP pathway [41,42]. This GIP-GIPR axis stimulates GH secretion [43], which might contribute to one of the causes of tumorigenesis in GHomas. The mechanism of how GIP expression is elevated in *GNAS* mutation-negative GHomas is still unclear and requires further studies.

As described above, although GHoma is a group of diseases with multiple cause of tumorigenesis, the tumor cause of which is mainly due to the activation of the cAMP pathway represented by somatic mutations in *GNAS*.

## 3. Pituitary Induction from Pluripotent Stem Cells

Human pluripotent stem cells (hPSCs) are classified into two types based on their establishment methods: embryonic stem cells (hESCs) [44] and induced pluripotent stem cells (iPSCs) [45]. The PSCs possess differentiation potentials into all three germ layer cell types. Many studies have reported divergent induction methods into various cells, including central nervous systems [46,47], cardiopulmonary systems [48,49], digestive tracts [50,51], and musculoskeletal systems [52,53]. Likewise, pituitary induction methods have also been established. The pituitary gland develops from the oral ectoderm, in contact with the adjacent hypothalamus, during embryonic development. During this process, interactions between the oral ectoderm and hypothalamus are key features of pituitary cell development, although the precise underlying mechanisms have not been fully understood. This differentiation process is tightly regulated in time and space by various growth factors and their downstream transcription factors. These factors include BMP-4, sonic hedgehog (SHH), fibroblast growth factor (FGF), and WNT signaling [54,55].

Recently, different methods to induce pituitary cells by mimicking these embryonic pituitary differentiation processes have been presented. We have shown, for the first time, pituitary inductions from mouse ESCs [56] by modifying our previous method for inducing hypothalamic organoids from mouse ESCs [57]. In the method, the minimization of exogenous patterning signals, such as growth factors, in the culture medium led to hypothalamic differentiation from mouse ESCs. We attempted to induce pituitary differentiation by reproducing the interaction between the hypothalamus and oral ectoderm in vitro. We found that increasing the number of cells in the organoid enhances endogenous BMP-4 singling, inducing oral ectoderm cells in the outer layer of the hypothalamic organoid. This simultaneous oral ectoderm and hypothalamus induction could recapitulate the interactions between these tissues, resulting in the self-organization of pituitary hormone-producing cells.

**Figure 1:**
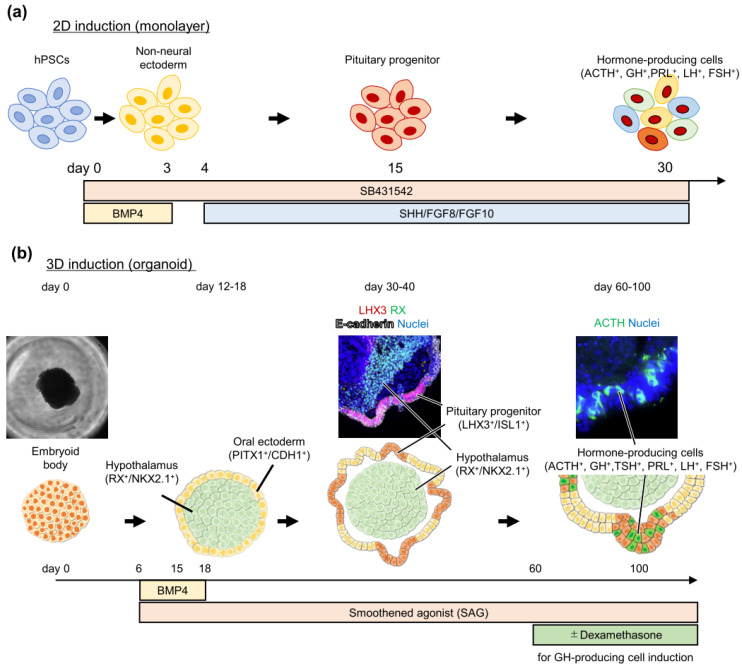
Two human pluripotent stem-cell induction methods into pituitary hormone-producing cells. (**a**) In the 2D induction method (monolayer culture), endogenous or exogenous BMP-4 signaling promotes non-neural ectodermal lineage. Then, treatment with surrounding factors that emerges in the physiological developmental process (sonic hedgehog (SHH) and fibroblast growth factor (FGF)-8 and -10) promotes the differentiation into pituitary progenitor and hormone-producing cells. (**b**) In the 3D induction method (organoid culture), oral ectoderm and hypothalamus are simultaneously induced in one cell aggregate. This simultaneous induction of two different tissues can recapitulate the tissue interactions between them, leading to the self-organization of pituitary progenitor cells and subsequent hormone-producing cells.

Studer’s group have first reported a pituitary induction method from hESCs using a two-dimensional monolayer culture [58] (Figure 1a). They modified their previous two-dimensional neural induction method, which is based on the concomitant inhibition of two SMAD-signaling pathways (BMP and TGF-β/Nodal signaling) named “dual SMAD inhibition” [46]. BMP inhibitor (Noggin) withdrawal allows for differentiation into non-neural placode lineage, and SHH and FGF signaling leads to pituitary cell differentiation [58,59]. They were able to induce all anterior pituitary hormone-producing cells, except for TSH-producing cells. Importantly, these pituitary cells induced from two- and three-dimensional cultures retain their physiological properties. They treated induced pituitary hormone-producing cells with their corresponding stimulant (CRH, stressin I, and urocortin for ACTH-producing cells; GHRH for GH-producing cells; GnRH analog for FSH -producing cells) and showed specific responses to the stimulant and secretion of pituitary hormone into the culture medium. We have also developed a three-dimensional pituitary induction method using hESCs and hiPSCs [60,61] (Figure 1b) with some modifications to the method for pituitary induction from mouse ESCs. Our method can induce all anterior pituitary hormone-producing cells, including ACTH, GH, TSH, PRL, and LH/FSH. These cells were also functional. Induced ACTH- and GH-producing cells responded specifically to CRH and GHRH, respectively, and secreted ACTH and GH. Furthermore, ACTH-producing cells showed feedback inhibition; treatment with hydrocortisone inhibited ACTH secretion. 

These two methods include BMP, SHH, and FGF signaling, which are important for physiological pituitary development [55], indicating that these methods recapitulate embryonic pituitary developmental process and yield natural pituitary hormone-producing cells. In fact, the transplantation of induced pituitary cells using both methods into a murine hypopituitarism model increased serum hormone levels and improved survival, confirming the functions of induced pituitary cells. These results suggest that induced pituitary cells are more similar to natural pituitary cells than rodent cell lines, such as AtT20 and GH3. Therefore, these technologies provide great opportunities to study human pituitary development and pituitary diseases. In addition, both methods can induce almost all anterior pituitary hormone-producing cells, suggesting that these methods can potentially model all functional pituitary adenomas.

Although both methods can induce functional pituitary hormone-producing cells, there are several differences. First, the culture duration for the induction is even shorter in two-dimensional culture. It takes 30 and 70 days for pituitary hormone-producing cell induction in two- and three-dimensional cultures, respectively. Second, hypothalamus cells are also induced simultaneously in the three-dimensional organoid. This simultaneous induction of the hypothalamus and pituitary recapitulates the functional regulation of the hypothalamus–pituitary axis [61]. Third, differentiation preference for pituitary hormone-producing cells is different. ACTH secretion is higher in three-dimensional culture, whereas GH secretion is higher in two-dimensional culture [58,59,60,61]. From these features, the suitable applications of each method appear to be different. The two-dimensional method may be better for regenerative cell therapy in patients with hypopituitarism, because of higher efficiency, a shorter induction period, and easier cell manufacturing. In contrast, for the disease modeling of pituitary disorders, the three-dimensional method might be superior because it can recapitulate the interaction between the hypothalamus and pituitary, which is required for the proper development in the embryonic period and the functional regulation in adults. Thus, this review envisions a three-dimensional culture in developing pituitary tumors in vitro models in the following sections.

### Pluripotent Stem Cell-Derived Pituitary Cell Application to Study Pituitary Diseases

One of the beneficial applications of hiPSCs is to study disease pathophysiology by inducing organs in which phenotypes emerge, particularly in genetic diseases. hiPSC-derived pituitary induction has been applied to study pituitary diseases. We established a hiPSC-based disease model for congenital hypopituitarism (CPH). CPH has various etiologies, and genetic abnormalities are important causes. Knockout mice have mainly been used to study the roles of responsible genes. However, there are significant differences among species, and their phenotypes are not necessarily identical to those in humans. In our study, patients-specific iPSCs were established from a patient with CPH, harboring a missense mutation in OTX2. OTX2 is a transcription factor involved in eye, brain, and pituitary gland developments. Although it is known that mutations in OTX2 result in CPH, the precise mechanisms have not been fully clarified. Three-dimensional models have revealed that OTX2 is a causative gene for the pathogenesis. OTX2 promotes pituitary progenitor cell differentiation by regulating FGF expression in the hypothalamus [62]. This study confirmed the validity of hiPSC-derived pituitary induction methods for the disease modeling of pituitary diseases.

We also developed iPSC-based disease models of the acquired pituitary disorder, anti-PIT-1 hypophysitis [63,64]. In the syndrome, PIT-1 lineage hormone-producing cells (GH-, TSH-, and PRL-producing cells) are specifically targeted by PIT-1 reactive cytotoxic T lymphocytes (CTLs) [64]. However, it remains unclear how the CTLs can recognize transcription factor PIT-1 that localize in nucleus. To establish the disease model, patient-derived iPSCs were induced into pituitary cells. We found that the PIT-1 epitope was processed in the antigen presentation pathway. In addition, patient’s iPSC-derived PIT-1 positive cells exhibited the co-localization of the PIT-1 epitope and HLA antigen on the cell surface. These results indicate that PIT-1-reactive CTLs can recognize the PIT1 epitope presented with the HLA antigen. Intriguingly, healthy control iPSC-derived PIT-1 positive cells also showed co-localization of the PIT-1 epitope and HLA antigen, indicating that the pathogenesis of the syndrome was not in the PIT-1 positive cells but in the CTLs [65]. These pituitary disease models clearly suggest the usefulness of hPSCs for the elucidation of the underlying mechanisms in pituitary disorders.

## 4. CRISPR/Cas9 Genome Editing

Genome editing using CRISPR/Cas technology has expanded rapidly and revolutionized biological studies. CRISPR/Cas9 systems were initially identified as adaptive immunity systems of bacteria and archaea against invasive foreign nucleic acids, such as bacteriophage infections [66]. This function is regulated by sequence-specific binding of CRISPR RNA (crRNA) to target DNA or RNA, with an additional requirement for a flanking DNA motif called the protospacer adjacent motif (PAM) in specific CRISPR systems. 

CRISPR/Cas9 is the most used CRISPR/Cas system for mammalian cells. crRNA, which is base-paired with trans-activating crRNA (tracrRNA), forms a two-RNA structure. This dual RNA complex recruits Cas9 to the target DNA locus and introduces double-strand breaks (DSB). This dual system was simplified to an engineered chimeric RNA molecule, single-guide RNA (sgRNA) [67]. These simple and easy-to-use systems are also functional in mammalian cells, thus introducing DSB into the target gene locus. This DSB can be utilized for genetic engineering by combining the different cellular mechanisms of DNA repair, including non-homologous end joining (NHEJ) and homology-directed repair (HDR) mechanisms. Cas9 cleavage and NHEJ-mediated re-ligation at the break point are repeated until an NHEJ error occurs. This error is typically a small insertion and deletion (indel), which may induce a frameshift mutation to generate gene knockouts. In addition, the desired genomic changes can be incorporated using HDR mechanisms. HDR copies a donor DNA strand into a region of the DSB. By exogenously delivering donor templates, such as plasmids and single-stranded DNA, with two homology arms on each side flanking the desired insertion, a precisely desired change can be introduced into the genome. These genome-editing technologies are now widely employed for various purposes, such as generating genetically engineered mice, studying the function of interesting genes and developing treatments for genetic disorders.

### 4.1. CRISPR/Cas9 Application to Study Tumor Pathophysiology

**Figure 2:**
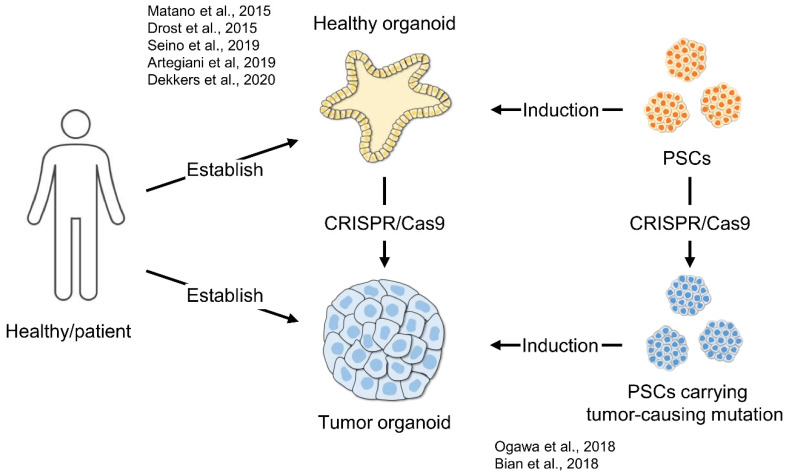
Tumor organoid model using CRISPR/Cas9 systems.

Several studies have used CRISPR/Cas9 genome-editing to establish in vitro cancer models by introducing cancer-causing mutations (Figure 2). In particular, organoid technologies have been used with CRISPR/Cas9 systems to study tumor formations in vitro [68]. The evidence support the idea that driver gene mutations such *USP8* and *GNAS* can be used to develop a pituitary tumor in vitro model.

Ogawa et al. developed a glioma model using human ESC-derived cerebral organoids. They introduced an *HRAS*^G12V^ oncogenic mutation into the *TP53* locus, which initiated cerebral organoid tumorigenesis, showing gene expression profiles that are consistent with mesenchymal subtype glioblastoma [69]. Bian et al. also established neoplastic cerebral organoid to study brain-tumor biology by introducing oncogenic mutations using the CRISPR/Cas9 systems. These engineered organoids are suitable for testing the effects of drugs in the context of specific gene mutations [70]. CRISPR-based mutagenesis is presently applied to normal tissue organoids, such as colon organoids, to study the adenoma–carcinoma progression in vitro. Matano et al. and Drost et al. established colorectal cancer models using normal human intestinal epithelium-derived organoids. Colorectal tumors have recurrent mutations in genes involved in the *WNT*, *MAPK*, *TGF-β*, *TP53*, and *PI3K* pathways. They introduced mutations in the tumor-suppressor genes, *APC*, *SMAD4*, and *TP53*, and the oncogenes *KRAS* and/or *PIK3CA* in the human intestinal organoids. Engineered organoids grow independently of niche factors in vitro and formed tumors after implantation into immunodeficient mice [71,72]. Artegiani et al. showed the tumor suppressor function of the deubiquitinating enzyme, BAP1 using CRISPR-engineered human liver organoids. They established human liver organoids with four major cholangiocarcinoma-related mutations (*TP53*, *PTEN*, *SMAD4*, and *NF1*). Notably, BAP1 loss causes the liver organoid to acquire malignant features during xenotransplantation [73]. Seino et al. engineered human pancreas organoids to carry *KRAS*, *CDKN2A*, *TP53*, and/or *SMAD4* mutations using CRISPR/Cas9 and investigated their engraftment potential in immunodeficient mice. They showed that WNT niche independence acquisition is important during pancreatic tumorigenesis [74]. Dekkers et al. established a breast cancer disease model by introducing cancer-related mutations into human breast organoids using CRISPR/Cas9. These engineered organoids responded to endocrine therapy or chemotherapy, suggesting their validity for drug testing and molecular profiles exploration [75]. These organoid techniques, combined with CRISPR/Cas9 genome-editing, provide novel platforms to study the process of human cancer progression in the laboratory. These technologies are also promising tools for drug discovery, precision medicine, and prevention. 

### 4.2. Hypothetical Strategies to Study Pituitary Adenoma Using Human Pluripotent Stem Cells

Although human pituitary organoids have not yet been reported, Cox et al. recently established pituitary organoids derived from mice. These mouse organoids originated from the Sox2+ pituitary progenitor cells and retained their stemness phenotype during culture expansion. However, the potential for differentiation into hormone-producing cells is limited even after subrenal transplantation [76]. Therefore, although organoids provide great opportunities to study pituitary stem cells, which have been poorly characterized, their application to disease models of pituitary hormone-producing cells is still difficult. Currently, pituitary induction technologies from hPSCs with CRISPR/Cas9 genome-editing are the best possible strategies for creating in vitro pituitary adenoma disease models. We present possible strategies to develop an hPSC-based in vitro model of ACTHoma and GHoma as follows.

**Figure 3:**
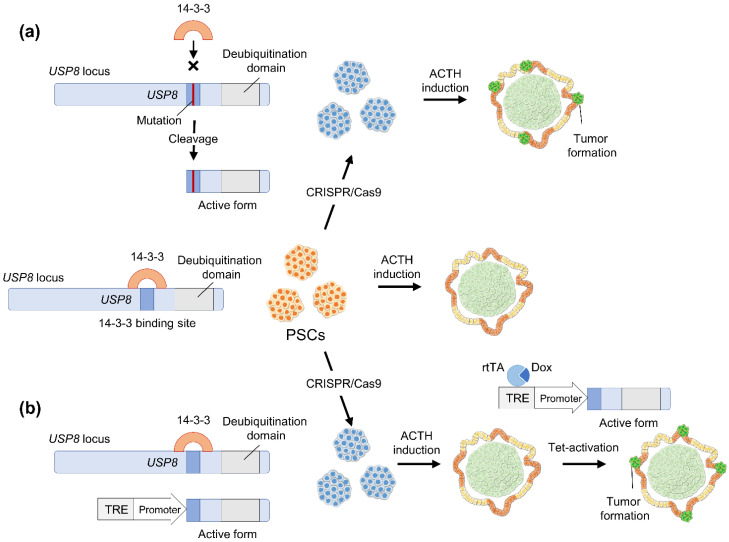
Schema of strategies to establish in vitro adrenocorticotropic hormone (ACTH)-producing adenoma (ACTHoma) model. (**a**) Gene mutation is introduced into human pluripotent stem cells (hPSCs) at the 14-3-3 binding site of USP8 using the CRISPR/Cas9 system. This mutation prevents 14-3-3 protein from binding to the site, enhancing proteolytic cleavage and catalytic activity of USP8. From the hPSCs, induction into ACTH-producing cells might form an ACTHoma-like tumor. (**b**) Inducible expression cassette (ex. tetracycline-inducible expression system) of the USP8 active form is introduced in hPSCs, leaving endogenous *USP8* locus intact. After induction into ACTH-producing cells, USP8 active-form expression is induced, which might form an ACTHoma-like tumor.

*USP8* mutations are a well-known cause of ACTHoma tumorigenesis. The simplest method to create a disease model is the introduction of point mutations within the 14-3-3 binding motif of USP8, which increases its deubiquitylation activity (Figure 3a). This approach can induce PSCs with *USP8* mutation into ACTH-producing cells to study ACTHomas. However, upregulating the deubiquitylation activity of USP8 during the differentiation process may interfere with pituitary cell induction itself. Therefore, USP8 mutant expression should be induced after ACTH-producing cell induction to mimic tumorigenesis in physiological ACTHoma. Accordingly, PSC-based ACTHoma models may also be established by incorporating a tet-ON system that induces mutant USP8 expression after ACTH-producing cell induction, leaving the the endogenous USP8 locus intact (Figure 3b).

**Figure 4:**
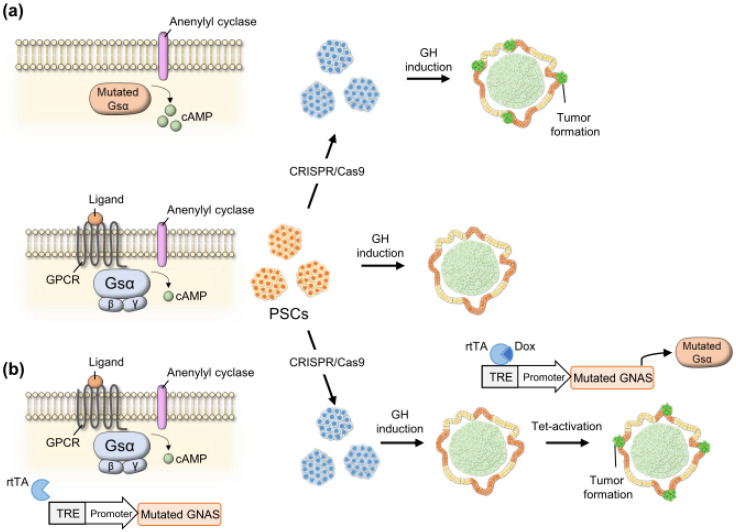
Schema of strategies to establish in vitro growth hormone (GH)-producing adenoma (GHoma) model. (**a**) A gain-of-function mutation is introduced into human pluripotent stem cells (hPSCs) at the guanine nucleotide-binding protein G(s) subunit alpha (*GNAS*) locus. The mutation activates Gsα and its effector adenylate cyclase, autonomously synthesizing cAMP. Induction into GH-producing cells from the hPSCs might form a GHoma-like tumor. (**b**) Inducible expression cassette (ex. tetracycline-inducible expression system) of Gsα active form is introduced in hPSCs, while leaving endogenous *GNAS* locus intact. After induction into GH-producing cells, Gsα active form expression is induced, which might form a GHoma-like tumor.

In GHomas, cAMP pathway upregulation is the primary cause of the tumorigenesis and *GNAS* is the target of interest. Several possible approaches are considered for introducing these genetic mutations. Similarly to ACTHoma, the simplest method is to incorporate gain-of-function mutations, such as p.R201C and p.R201H, into the endogenous *GNAS* locus (Figure 4a). However, because the cAMP pathway is involved in various physiological functions, constitutive cAMP pathway activation may also interfere with the induction process. Therefore, the inducible expression of active GNAS after GH-producing cell induction might also be advantageous to model GHomas (Figure 4b). Tumorigenic mechanisms of other GHoma causative genes, such as MEN1, AIP, MEN4, have not been fully understood. Knockout iPSCs for these genes might be helpful to analyze the underlying mechanisms that cause GHoma.

In pituitary adenomas, there are several clinical and tumor biological issues that have not been fully clarified. For example, response rates of current medical therapies for ACTHoma and GHoma are inadequate. In addition, pituitary adenomas show great phenotypic variations such as invasiveness and response to medical treatment, even when these tumors have the same causative gene mutation. The factors that define these phenotypes have not been elucidated. Establishing the hPSC-based pituitary adenoma model will support the developments of new medical treatments, precision medicines, and studies of the mechanisms underlying pituitary tumorigenesis and pathophysiology.

## 5. Conclusions

In this review, we described pituitary adenoma pathogenesis, pituitary induction methods using hPSCs, and CRISPR/Cas9 applications to study cancer pathophysiology. These facts clearly suggest that combining hPSC-derived pituitary induction methods and CRISPR/Cas9 technologies may develop novel pituitary adenoma disease models. There will be significant advantages with respect to issues such as species and phenotypic differences compared to rodent cell lines and tumor model mice. The lack of reliable human pituitary cell lines is a significant obstacle in pituitary adenoma research. Several pituitary cell lines, such as AtT20 and GH3, have been derived from rodent tumor tissues. However, these cell lines have already lost some physiological features and are different from the human pituitary gland. These cell line characteristics and species differences impede the application of human pituitary adenoma models. Therefore, hPSC-derived pituitary cells may replace these research materials.

Although the pituitary research using hPSCs is still limited, this in vitro model will become a powerful tool for studying normal pituitary development and diseases. This method allows us the examination of the stepwise differentiation steps of PSCs into pituitary hormone-producing cells in vitro. These model systems have also been utilized to establish models of congenital diseases, such as congenital hypopituitarism. This method also provides opportunities to model pituitary adenoma. If hPCS-based pituitary adenoma model is developed, tumor development can be observed longitudinally using this in vitro model, leading to the investigation of pituitary adenoma progression. In addition, analyzing the pathogenesis and characteristics and developing novel therapeutic agents will be worthwhile. Furthermore, the culture conditions might also be beneficial for establishing human tissue-derived pituitary organoids and patient-derived pituitary adenoma organoids, providing tremendous opportunities for precision medicine.

In summary, recent progress in the hPSC-based induction methods, genome-editing, and organoid technologies will undoubtedly allow the development of novel disease models for pituitary adenomas. These models will provoke drug discovery and pathophysiological understanding, leading to clinical breakthroughs in the management of pituitary adenoma patients.

## Data Availability

Data are contained within the article.

## References

[1] Herman V., Fagin J., Gonsky R., Kovacs K., Melmed S. (1990). Clonal origin of pituitary adenomas. J. Clin. Endocrinol. Metab..

[2] Ostrom Q.T., Gittleman H., Liao P., Rouse C., Chen Y., Dowling J., Wolinsky Y., Kruchko C., Barnholtz-Sloan J. (2014). CBTRUS statistical report: Primary brain and central nervous system tumors diagnosed in the United States in 2007-2011. Neuro Oncol..

[3] Dallapiazza R.F., Oldfield E.H., Jane J.A. (2015). Surgical management of Cushing’s disease. Pituitary.

[4] Amlashi F.G., Tritos N.A. (2016). Thyrotropin-secreting pituitary adenomas: Epidemiology, diagnosis, and management. Endocrine.

[5] Fleseriu M., Biller B.M.K., Freda P.U., Gadelha M.R., Giustina A., Katznelson L., Molitch M.E., Samson S.L., Strasburger C.J., van der Lely A.J. (2021). A Pituitary Society update to acromegaly management guidelines. Pituitary.

[6] Fleseriu M., Auchus R., Bancos I., Ben-Shlomo A., Bertherat J., Biermasz N.R., Boguszewski C.L., Bronstein M.D., Buchfelder M., Carmichael J.D. (2021). Consensus on diagnosis and management of Cushing’s disease: A guideline update. Lancet Diabetes Endocrinol..

[7] Ono M., Miki N., Kawamata T., Makino R., Amano K., Seki T., Kubo O., Hori T., Takano K. (2008). Prospective study of high-dose cabergoline treatment of prolactinomas in 150 patients. J. Clin. Endocrinol. Metab..

[8] Gadelha M.R., Bronstein M.D., Brue T., Coculescu M., Fleseriu M., Guitelman M., Pronin V., Raverot G., Shimon I., Lievre K.K. (2014). Pasireotide versus continued treatment with octreotide or lanreotide in patients with inadequately controlled acromegaly (PAOLA): A randomised, phase 3 trial. Lancet Diabetes Endocrinol..

[9] Caron P.J., Bevan J.S., Petersenn S., Flanagan D., Tabarin A., Prévost G., Maisonobe P., Clermont A., PRIMARYS Investigators (2014). Tumor Shrinkage With Lanreotide Autogel 120 mg as Primary Therapy in Acromegaly: Results of a Prospective Multicenter Clinical Trial. J. Clin. Endocrinol. Metab..

[10] Clayton R.N., Raskauskiene D., Reulen R.C., Jones P.W. (2011). Mortality and morbidity in Cushing’s disease over 50 years in Stoke-on-Trent, UK: Audit and meta-analysis of literature. J. Clin. Endocrinol. Metab..

[11] Larsson C., Skogseid B., Oberg K., Nakamura Y., Nordenskjöld M. (1988). Multiple endocrine neoplasia type 1 gene maps to chromosome 11 and is lost in insulinoma. Nature.

[12] Pellegata N.S., Quintanilla-Martinez L., Siggelkow H., Samson E., Bink K., Höfler H., Fend F., Graw J., Atkinson M.J. (2006). Germ-line mutations in p27Kip1 cause a multiple endocrine neoplasia syndrome in rats and humans. Proc. Natl. Acad. Sci. USA.

[13] Daly A.F., Jaffrain-Rea M.L., Ciccarelli A., Valdes-Socin H., Rohmer V., Tamburrano G., Borson-Chazot C., Estour B., Ciccarelli E., Brue T. (2006). Clinical characterization of familial isolated pituitary adenomas. J. Clin. Endocrinol. Metab..

[14] Yaneva M., Vandeva S., Zacharieva S., Daly A.F., Beckers A. (2010). Genetics of Cushing’s syndrome. Neuroendocrinology.

[15] Ma Z.Y., Song Z.J., Chen J.H., Wang Y.F., Li S.Q., Zhou L.F., Mao Y., Li Y.M., Hu R.G., Zhang Z.Y. (2015). Recurrent gain-of-function USP8 mutations in Cushing’s disease. Cell Res..

[16] Reincke M., Sbiera S., Hayakawa A., Theodoropoulou M., Osswald A., Beuschlein F., Meitinger T., Mizuno-Yamasaki E., Kawaguchi K., Saeki Y. (2015). Mutations in the deubiquitinase gene USP8 cause Cushing’s disease. Nat. Genet..

[17] Perez-Rivas L.G., Theodoropoulou M., Ferraù F., Nusser C., Kawaguchi K., Stratakis C.A., Faucz F.R., Wildemberg L.E., Assié G., Beschorner R. (2015). The Gene of the Ubiquitin-Specific Protease 8 Is Frequently Mutated in Adenomas Causing Cushing’s Disease. J. Clin. Endocrinol. Metab..

[18] Hayashi K., Inoshita N., Kawaguchi K., Ibrahim Ardisasmita A., Suzuki H., Fukuhara N., Okada M., Nishioka H., Takeuchi Y., Komada M. (2016). The USP8 mutational status may predict drug susceptibility in corticotroph adenomas of Cushing’s disease. Eur. J. Endocrinol..

[19] Theodoropoulou M., Arzberger T., Gruebler Y., Jaffrain-Rea M.L., Schlegel J., Schaaf L., Petrangeli E., Losa M., Stalla G.K., Pagotto U. (2004). Expression of epidermal growth factor receptor in neoplastic pituitary cells: Evidence for a role in corticotropinoma cells. J. Endocrinol..

[20] Fukuoka H., Cooper O., Ben-Shlomo A., Mamelak A., Ren S.G., Bruyette D., Melmed S. (2011). Egfr as a therapeutic target for human, canine, and mouse ACTH-secreting pituitary adenomas. J. Clin. Investig..

[21] Chen J., Jian X., Deng S., Ma Z., Shou X., Shen Y., Zhang Q., Song Z., Li Z., Peng H. (2018). Identification of recurrent USP48 and BRAF mutations in Cushing’s disease. Nat. Commun..

[22] Sbiera S., Perez-Rivas L.G., Taranets L., Weigand I., Flitsch J., Graf E., Monoranu C.M., Saeger W., Hagel C., Honegger J. (2019). Driver mutations in USP8 wild-type Cushing’s disease. Neuro Oncol..

[23] Lidhar K., Korbonits M., Jordan S., Khalimova Z., Kaltsas G., Lu X., Clayton R.N., Jenkins P.J., Monson J.P., Besser G.M. (1999). Low expression of the cell cycle inhibitor p27Kip1 in normal corticotroph cells, corticotroph tumors, and malignant pituitary tumors. J. Clin. Endocrinol. Metab..

[24] Jordan S., Lidhar K., Korbonits M., Lowe D.G., Grossman A.B. (2000). Cyclin D and cyclin E expression in normal and adenomatous pituitary. Eur. J. Endocrinol..

[25] Roussel-Gervais A., Couture C., Langlais D., Takayasu S., Balsalobre A., Rueda B.R., Zukerberg L.R., Figarella-Branger D., Brue T., Drouin J. (2016). The Cables1 Gene in Glucocorticoid Regulation of Pituitary Corticotrope Growth and Cushing Disease. J. Clin. Endocrinol. Metab..

[26] Melmed S. (2006). Acromegaly. N. Engl. J. Med..

[27] Matsumoto R., Izawa M., Fukuoka H., Iguchi G., Odake Y., Yoshida K., Bando H., Suda K., Nishizawa H., Takahashi M. (2016). Genetic and clinical characteristics of japanese patients with sporadic somatotropinoma. Endocr. J..

[28] Mendoza V., Sosa E., Espinosa-de-los-Monteros A.L., Salcedo M., Guinto G., Cheng S., Sandoval C., Mercado M. (2005). Gspα mutations in Mexican patients with acromegaly: Potential impact on long term prognosis. Growth Horm. IGF Res..

[29] Landis C.A., Masters S.B., Spada A., Pace A.M., Bourne H.R., Vallar L. (1989). GTPase inhibiting mutations activate the α chain of Gs and stimulate adenylyl cyclase in human pituitary tumours. Nature.

[30] Freda P., Chung W., Matsuoka N., Walsh J., Kanibir M.N., Kleinman G., Wang Y., Bruce J., Post K. (2007). Analysis of GNAS mutations in 60 growth hormone secreting pituitary tumors: Correlation with clinical and pathological characteristics and surgical outcome based on highly sensitive GH and IGF-I criteria for remission. Pituitary.

[31] Vallar L., Spada A., Giannattasio G. (1987). Altered Gs and adenylate cyclase activity in human GH-secreting pituitary adenomas. Nature.

[32] Chandrasekharappa S.C., Guru S.C., Manickam P., Olufemi S.E., Collins F.S., Emmert-Buck M.R., Debelenko L.V., Zhuang Z., Lubensky I.A., Liotta L.A. (1997). Positional cloning of the gene for multiple endocrine neoplasia-type 1. Science.

[33] Kirschner L.S., Carney J.A., Pack S.D., Taymans S.E., Giatzakis C., Cho Y.S., Cho-Chung Y.S., Stratakis C.A. (2000). Mutations of the gene encoding the protein kinase A type I-alpha regulatory subunit in patients with the Carney complex. Nat. Genet..

[34] Vierimaa O., Georgitsi M., Lehtonen R., Vahteristo P., Kokko A., Raitila A., Tuppurainen K., Ebeling T.M.L., Salmela P.I., Paschke R. (2006). Pituitary Adenoma Predisposition Caused by Germline Mutations in the AIP Gene. Science.

[35] Trivellin G., Daly A.F., Faucz F.R., Yuan B., Rostomyan L., Larco D.O., Schernthaner-Reiter M.H., Szarek E., Leal L.F., Caberg J.H. (2014). Gigantism and acromegaly due to Xq26 microduplications and GPR101 mutation. N. Engl. J. Med..

[36] Bogusławska A., Korbonits M. (2021). Genetics of Acromegaly and Gigantism. J. Clin. Med..

[37] Valimaki N., Demir H., Pitkanen E., Kaasinen E., Karppinen A., Kivipelto L., Schalin-Jantti C., Aaltonen L.A., Karhu A. (2015). Whole-Genome Sequencing of Growth Hormone (GH)-Secreting Pituitary Adenomas. J. Clin. Endocrinol. Metab..

[38] Neou M., Villa C., Armignacco R., Jouinot A., Raffin-Sanson M.-L., Septier A., Letourneur F., Diry S., Diedisheim M., Izac B. (2020). Pangenomic Classification of Pituitary Neuroendocrine Tumors. Cancer Cell.

[39] Occhi G., Losa M., Albiger N., Trivellin G., Regazzo D., Scanarini M., Monteserin-Garcia J.L., Frohlich B., Ferasin S., Terreni M.R. (2011). The glucose-dependent insulinotropic polypeptide receptor is overexpressed amongst GNAS1 mutation-negative somatotropinomas and drives growth hormone (GH)-promoter activity in GH3 cells. J. Neuroendocrinol..

[40] Umahara M., Okada S., Ohshima K., Mori M. (2003). Glucose-dependent insulinotropic polypeptide induced growth hormone secretion in acromegaly. Endocr. J..

[41] Volz A., Goke R., Lankat-Buttgereit B., Fehmann H.C., Bode H.P., Goke B. (1995). Molecular cloning, functional expression, and signal transduction of the GIP-receptor cloned from a human insulinoma. FEBS Lett..

[42] Gremlich S., Porret A., Hani E.H., Cherif D., Vionnet N., Froguel P., Thorens B. (1995). Cloning, functional expression, and chromosomal localization of the human pancreatic islet glucose-dependent insulinotropic polypeptide receptor. Diabetes.

[43] Regazzo D., Losa M., Albiger N.M., Terreni M.R., Vazza G., Ceccato F., Emanuelli E., Denaro L., Scaroni C., Occhi G. (2017). The GIP/GIPR axis is functionally linked to gh-secretion increase in a significant proportion of gsp− somatotropinomas. Eur. J. Endocrinol..

[44] Thomson J.A., Itskovitz-Eldor J., Shapiro S.S., Waknitz M.A., Swiergiel J.J., Marshall V.S., Jones J.M. (1998). Embryonic Stem Cell Lines Derived from Human Blastocysts. Science.

[45] Takahashi K., Tanabe K., Ohnuki M., Narita M., Ichisaka T., Tomoda K., Yamanaka S. (2007). Induction of pluripotent stem cells from adult human fibroblasts by defined factors. Cell.

[46] Chambers S.M., Fasano C.A., Papapetrou E.P., Tomishima M., Sadelain M., Studer L. (2009). Highly efficient neural conversion of human ES and iPS cells by dual inhibition of SMAD signaling. Nat. Biotechnol..

[47] Lancaster M.A., Renner M., Martin C.-A., Wenzel D., Bicknell L.S., Hurles M.E., Homfray T., Penninger J.M., Jackson A.P., Knoblich J.A. (2013). Cerebral organoids model human brain development and microcephaly. Nature.

[48] Gotoh S., Ito I., Nagasaki T., Yamamoto Y., Konishi S., Korogi Y., Matsumoto H., Muro S., Hirai T., Funato M. (2014). Generation of Alveolar Epithelial Spheroids via Isolated Progenitor Cells from Human Pluripotent Stem Cells. Stem Cell Rep..

[49] Burridge P.W., Matsa E., Shukla P., Lin Z.C., Churko J.M., Ebert A.D., Lan F., Diecke S., Huber B., Mordwinkin N.M. (2014). Chemically defined generation of human cardiomyocytes. Nat. Methods.

[50] Spence J.R., Mayhew C.N., Rankin S.A., Kuhar M.F., Vallance J.E., Tolle K., Hoskins E.E., Kalinichenko V.V., Wells S.I., Zorn A.M. (2011). Directed differentiation of human pluripotent stem cells into intestinal tissue in vitro. Nature.

[51] Crespo M., Vilar E., Tsai S.-Y., Chang K., Amin S., Srinivasan T., Zhang T., Pipalia N.H., Chen H.J., Witherspoon M. (2017). Colonic organoids derived from human induced pluripotent stem cells for modeling colorectal cancer and drug testing. Nat. Med..

[52] Hosoyama T., McGivern J.V., Van Dyke J.M., Ebert A.D., Suzuki M. (2014). Derivation of myogenic progenitors directly from human pluripotent stem cells using a sphere-based culture. Stem Cells Transl. Med..

[53] Oldershaw R.A., Baxter M.A., Lowe E.T., Bates N., Grady L.M., Soncin F., Brison D.R., Hardingham T.E., Kimber S.J. (2010). Directed differentiation of human embryonic stem cells toward chondrocytes. Nat. Biotechnol..

[54] Matsumoto R., Takahashi Y. (2020). Human pituitary development and application of iPSCs for pituitary disease. Cell. Mol. Life Sci..

[55] Zhu X., Gleiberman A.S., Rosenfeld M.G. (2007). Molecular Physiology of Pituitary Development: Signaling and Transcriptional Networks. Physiol. Rev..

[56] Suga H., Kadoshima T., Minaguchi M., Ohgushi M., Soen M., Nakano T., Takata N., Wataya T., Muguruma K., Miyoshi H. (2011). Self-formation of functional adenohypophysis in three-dimensional culture. Nature.

[57] Wataya T., Ando S., Muguruma K., Ikeda H., Watanabe K., Eiraku M., Kawada M., Takahashi J., Hashimoto N., Sasai Y. (2008). Minimization of exogenous signals in ES cell culture induces rostral hypothalamic differentiation. Proc. Natl. Acad. Sci. USA.

[58] Dincer Z., Piao J., Niu L., Ganat Y., Kriks S., Zimmer B., Shi S.H., Tabar V., Studer L. (2013). Specification of functional cranial placode derivatives from human pluripotent stem cells. Cell Rep..

[59] Zimmer B., Piao J., Ramnarine K., Tomishima M.J., Tabar V., Studer L. (2016). Derivation of Diverse Hormone-Releasing Pituitary Cells from Human Pluripotent Stem Cells. Stem Cell Rep..

[60] Ozone C., Suga H., Eiraku M., Kadoshima T., Yonemura S., Takata N., Oiso Y., Tsuji T., Sasai Y. (2016). Functional anterior pituitary generated in self-organizing culture of human embryonic stem cells. Nat. Commun..

[61] Kasai T., Suga H., Sakakibara M., Ozone C., Matsumoto R., Kano M., Mitsumoto K., Ogawa K., Kodani Y., Nagasaki H. (2020). Hypothalamic Contribution to Pituitary Functions Is Recapitulated In Vitro Using 3D-Cultured Human iPS Cells. Cell Rep..

[62] Matsumoto R., Suga H., Aoi T., Bando H., Fukuoka H., Iguchi G., Narumi S., Hasegawa T., Muguruma K., Ogawa W. (2020). Congenital pituitary hypoplasia model demonstrates hypothalamic OTX2 regulation of pituitary progenitor cells. J. Clin. Invest.

[63] Yamamoto M., Iguchi G., Takeno R., Okimura Y., Sano T., Takahashi M., Nishizawa H., Handayaningshi A.E., Fukuoka H., Tobita M. (2011). Adult combined GH, prolactin, and TSH deficiency associated with circulating PIT-1 antibody in humans. J. Clin. Investig..

[64] Bando H., Iguchi G., Fukuoka H., Yamamoto M., Hidaka-Takeno R., Okimura Y., Matsumoto R., Suda K., Nishizawa H., Takahashi M. (2014). Involvement of PIT-1-reactive cytotoxic T lymphocytes in anti-PIT-1 antibody syndrome. J. Clin. Endocrinol. Metab..

[65] Kanie K., Bando H., Iguchi G., Muguruma K., Matsumoto R., Hidaka-Takeno R., Okimura Y., Yamamoto M., Fujita Y., Fukuoka H. (2019). Pathogenesis of anti-PIT-1 antibody syndrome: PIT-1 presentation by HLA Class I on Anterior Pituitary Cells. J. Endocr. Soc..

[66] Barrangou R., Fremaux C., Deveau H., Richards M., Boyaval P., Moineau S., Romero D.A., Horvath P. (2007). CRISPR provides acquired resistance against viruses in prokaryotes. Science.

[67] Jinek M., Chylinski K., Fonfara I., Hauer M., Doudna J.A., Charpentier E. (2012). A programmable Dual-RNA-guided DNA Endonuclease in Adaptive Bacterial Immunity. Science.

[68] Tuveson D., Clevers H. (2019). Cancer modeling meets human organoid technology. Science.

[69] Ogawa J., Pao G.M., Shokhirev M.N., Verma I.M. (2018). Glioblastoma Model Using Human Cerebral Organoids. Cell Rep..

[70] Bian S., Repic M., Guo Z., Kavirayani A., Burkard T., Bagley J.A., Krauditsch C., Knoblich J.A. (2018). Genetically engineered cerebral organoids model brain tumor formation. Nat. Methods.

[71] Matano M., Date S., Shimokawa M., Takano A., Fujii M., Ohta Y., Watanabe T., Kanai T., Sato T. (2015). Modeling colorectal cancer using CRISPR-Cas9-mediated engineering of human intestinal organoids. Nat. Med..

[72] Drost J., van Jaarsveld R.H., Ponsioen B., Zimberlin C., van Boxtel R., Buijs A., Sachs N., Overmeer R.M., Offerhaus G.J., Begthel H. (2015). Sequential cancer mutations in cultured human intestinal stem cells. Nature.

[73] Artegiani B., van Voorthuijsen L., Lindeboom R.G.H., Seinstra D., Heo I., Tapia P., López-Iglesias C., Postrach D., Dayton T., Oka R. (2019). Probing the Tumor Suppressor Function of BAP1 in CRISPR-Engineered Human Liver Organoids. Cell Stem Cell.

[74] Seino T., Kawasaki S., Shimokawa M., Tamagawa H., Toshimitsu K., Fujii M., Ohta Y., Matano M., Nanki K., Kawasaki K. (2018). Human Pancreatic Tumor Organoids Reveal Loss of Stem Cell Niche Factor Dependence during Disease Progression. Cell Stem Cell.

[75] Dekkers J.F., Whittle J.R., Vaillant F., Chen H.R., Dawson C., Liu K., Geurts M.H., Herold M.J., Clevers H., Lindeman G.J. (2020). Modeling Breast Cancer Using CRISPR-Cas9-Mediated Engineering of Human Breast Organoids. J. Natl. Cancer Inst..

[76] Cox B., Laporte E., Vennekens A., Kobayashi H., Nys C., Van Zundert I., Uji I.H., Vercauteren Drubbel A., Beck B., Roose H. (2019). Organoids from pituitary as a novel research model toward pituitary stem cell exploration. J. Endocrinol..

